# Assessment of microneedle array insertion into skin using Raman spectroscopic techniques

**DOI:** 10.1007/s13346-025-02041-1

**Published:** 2026-01-13

**Authors:** Rezvan Jamaledin, Panagiota Zarmpi, Adrián M. Alambiaga-Caravaca, Vasundhara Tyagi, Qonita Kurnia Anjani, Eneko Larrañeta, Ryan F. Donnelly, Natalie A. Belsey, Richard H. Guy, M. Begoña Delgado-Charro

**Affiliations:** 1 Department of Life Sciences, University of Bath, Bath, BA2 7AY UK; 2 Present Address: University of Surrey, School of Chemistry & Chemical Engineering, Guildford, GU2 7XH UK; 3 National Physical Laboratory, Teddington, TW11 0LW UK; 4 School of Pharmacy, Queen’s University Belfast, Medical Biology Centre, Belfast, BT9 7BL UK

**Keywords:** Microneedles, Raman spectroscopy, Stimulated Raman scattering microscopy, Skin, Microneedle insertion

## Abstract

Microneedle (MN) arrays bypass the skin’s stratum corneum barrier to deliver drugs directly into the viable tissue. The skin disposition of three types of MNs—dissolvable, degradable and hydrogel-forming, fabricated using different polymers—have been evaluated post-treatment with examples of these MN arrays and then examined by confocal Raman spectroscopy and stimulated Raman scattering (SRS) microscopy. The presence of the polymers was assessed from their characteristic Raman signals. SRS image mosaics were acquired to survey and visualise larger areas of the skin surface. After MN insertion, the skin's spectrum was acquired using confocal Raman spectroscopy at the surface, and at nominal depths of 50 µm, 100 µm, and 150 µm. For dissolvable and degradable MNs, Raman signals from the constituent polymers were detectable in the skin. However, the polymer used to form the hydrogel MNs was not detectable under the experimental conditions used. SRS confirmed that the MN arrays penetrated the skin in a reasonably uniform manner. In summary, polymeric MN insertion into the skin has been visualised using confocal Raman spectroscopy and SRS microscopy. Together, these techniques have the potential to shed light on the spatial and temporal skin disposition of the constituent MN polymers used.

**Graphical abstract:**

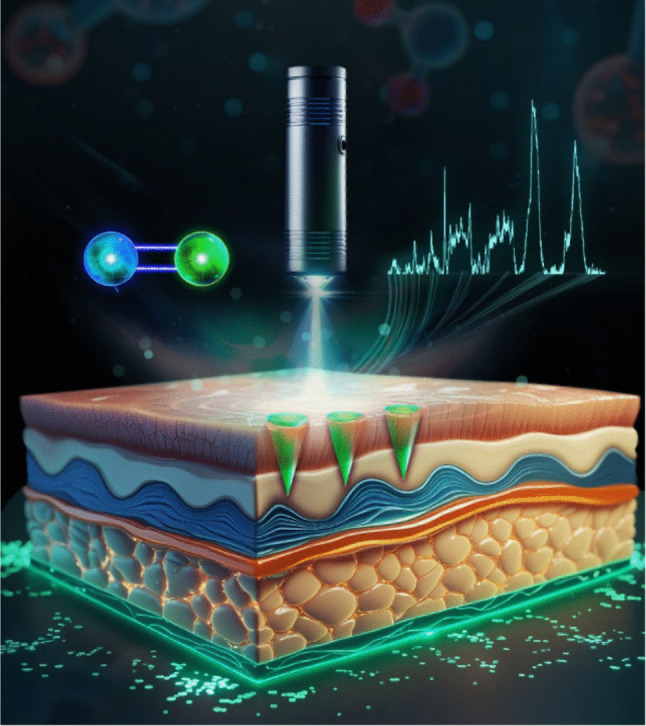

**Supplementary Information:**

The online version contains supplementary material available at 10.1007/s13346-025-02041-1.

## Introduction

The use of microneedles (MNs) to circumvent the skin’s principal barrier – the *stratum corneum* – to topical/transdermal drug delivery represents a well-established field of research [1]. An outstanding issue, however, that must be addressed before translation of this technology into safe and effective medical products, is to characterise the disposition of the constitutive polymers of the MNs post-insertion into the skin. Broadly speaking, MNs can be categorised into dissolvable, degradable and hydrogel-forming forms [1–4]. The former two are composed of polymers that, once pushed into the skin, are designed to either dissolve (dissolvable) — releasing the drug as the intact polymer dissolves into the interstitial fluid — or be chemically broken down into biocompatible monomers (degradable), with kinetics tailored to achieve the desired controlled drug release. The hydrogel-forming MNs, employ cross-linked polymers that absorb interstitial fluid post-insertion and swell to form gel-like structures from which controllable drug input can again be achieved at the desired rate.

Physical assessment of the skin penetration efficiency of MN arrays has been widely reported, as this impacts directly on the success of drug delivery [5, 6]. Several factors, including insertion force and skin elasticity, have been shown to significantly influence penetration efficiency [7]. Among the methods used to assess a MN array’s ability and uniformity to puncture the skin are computed tomography scanning and, in particular, optical coherence tomography (OCT) [8, 9]. However, while these tools can visualise the entry (or not) of MNs into the skin, they are unable to detect drug release or track the fate and disposition of the polymer(s) from which the MNs are made. Fluorescence imaging has provided some insight, but it is limited to inherently fluorescent compounds or those requiring fluorescent tagging, which can alter their physicochemical properties [10, 11].

Raman spectroscopy, on the other hand, has proven to be a valuable, label-free option with which to examine the disposition of certain topical drugs and formulation ingredients in the skin post-application of topical products [12, 13]. In one approach, confocal Raman spectroscopy interrogates the skin as a function of depth following the judicious selection of a molecular vibrational signal that lacks interference from both those emanating from endogenous skin species and from formulation excipients that also penetrate the barrier [14]. A more sophisticated approach is stimulated Raman scattering (SRS) microscopy, a 2-photon method involving the coherent stimulation of a specific molecular vibration unique to a single chemical functional group and enabling rapid image acquisition, and the ability to interrogate and image over relatively large (compared to confocal Raman) skin areas. By tuning the frequency difference between the two laser beams, different spectroscopic signals unique to different chemical species can be tracked sequentially and, in combination with second harmonic generation (SHG) or two-photon fluorescence, other molecules (e.g., collagen) can be visualised simultaneously [11, 15].

This study aimed to evaluate the depth-dependent distribution and spatial penetration of polymer constituents from dissolvable, degradable, and hydrogel-forming MN arrays in porcine skin ex vivo. To achieve this, confocal Raman spectroscopy and SRS microscopy were used individually and in combination to track the polymers' fate and provide three-dimensional visualisation of their penetration across a broader skin surface area.

## Materials and methods

### Materials

Gantrez® S-97 BF, copolymer of methyl vinyl ether and maleic acid (PMVE/MA, molecular weight = 1,200 kDa), poly(vinylpyrrolidone) K29/32 (PVP, molecular weight = 58 kDa) and Viatel™ DLG 7503 A/E, poly(lactic-co-glycolic acid) (PLGA), a 75:25 molar ratio of lactide: glycolide (molecular weight = 15–40 kDa), were provided by Ashland (Kidderminster, UK). Poly(vinyl alcohol) (PVA) (MW 9–10 kDa, 80% hydrolysed) and poly(vinyl pyrrolidone) (PVP) K90 (MW 360 kDa) and poly(ethylene glycol) (PEG, molecular weight = 10 kDa) were purchased from Sigma-Aldrich (Gillingham, UK). Glycerol was sourced from VWR (Solon, OH, USA). Imaging spacers of double-sided adhesive tape were purchased from Grace Bio-Labs (SecureSeal™, Bend, OR, USA). Parafilm™ was acquired from Bemis Company, Inc. (Neenah, WI, USA). Fresh abdominal porcine skin was obtained from a tissue supplier and dermatomed (Zimmer®, Hudson, OH, USA) to a nominal thickness of 750 μm, within 48 h post-slaughter. Visually obvious hairs were trimmed with scissors (avoiding contact between scissors and skin) and the tissue was stored at −20 °C until it was thawed shortly before use.

### Preparation of MN arrays

Dissolving MNs were prepared using a previously described casting method [16]. A 50 mg aliquot of an aqueous mixture of 20% w/w polyvinyl alcohol (PVA) and 20% w/w polyvinylpyrrolidone (PVP) K29/32 was poured into a poly(dimethylsiloxane) (PDMS) mould and subjected to a pressure of 5 bar (0.5 MPa) for 5 min. The mould was then centrifuged (Eppendorf® 5804 series centrifuge, Fisher Scientific, Loughborough, UK) at 5000 rpm for 10 min at 4 °C. Next, a silicone ring was glued onto the mould using the PVA solution and allowed to dry for 24 h at room temperature. The second, baseplate layer was then created from 500 μL of 30% w/w PVP K90 and 1.5% glycerol, which was centrifuged and dried for 24 h at room temperature. The sidewalls of the baseplate were trimmed using scissors and transferred to an oven at 37 °C for a final drying period of 24 h. The resulting dissolving MNs comprised a 16 × 16 array (over 0.5 cm^2^) with a height of 850 µm and baseplate width of 300 µm.

Degradable MNs were made by first dissolving 0.1 g of poly(lactic-co-glycolic acid (PLGA) in 1 mL of dimethyl sulphoxide (DMSO) using vortex mixing (Vortex™, Fisons Scientific Equipment, Loughborough, UK) and sonication (U500H ultrasonic cleaning bath, Ultrawave, Ltd., Cardiff, UK) for 5 min [17]. A 50 mg aliquot of this solution was poured into an identical PDMS mould as used for the dissolving MNs and similarly subjected to a pressure of 5 bar (0.5 MPa) for 5 min. Excess formulation was scraped off before drying overnight at room temperature. Then, 500 µL of a mixture of 20% w/w PVA and 20% w/w PVP K29/32 was used to cover the mould, centrifuged, and dried for 24 h at room temperature. Finally, and as before, the sidewalls of this baseplate were trimmed and then transferred to an oven at 37 °C for a final drying period of 24 h.

As previously reported [18], hydrogel-forming MNs were prepared using 20% w/w Gantrez® and 7.5% w/w polyethylene glycol (PEG) 10,000. The PEG was dissolved in a minimal volume of deionised water, added to Gantrez® S-97 and mixed. 500 mg of the formulation were cast into laser-ablated silicon templates (Blueacre Technology Ltd., Dundalk, Ireland) with mould dimensions of 600 µm height, 350 µm base, and an array of 11 × 11 MNs over an area of 1 cm^2^. The moulds were centrifuged at 3,500 rpm for 15 min and dried at room temperature for 48 h. The baseplate sidewalls were trimmed and then transferred to an oven at 80 °C for crosslinking for 24 h to form the hydrogel-forming matrix structure.

### In vitro MN insertion studies

To improve MN insertion repeatability, skin samples were secured on a plastic chopping board with thumbtacks. MN arrays were inserted into the skin using a plunger (height: 7 cm, diameter: 1 cm) that applied a consistent downward force. The plunger was manually aligned and held in position for 1 min to ensure uniform application across samples. The skin was then mounted in a standard, static Franz cell of 2.01 cm^2^ diffusion area (PermeGear, Hellertown, PA) with the MN treated area placed centrally on the tissue, which was clamped between the donor and receptor compartments; the latter was filled with 7.4 mL of phosphate-buffer saline (PBS) at pH 7.4. The donor compartment and sampling arm of the receptor compartment were occluded with parafilm, and the diffusion cell was then kept at 32 °C in an oven for 1 h. At this point, the baseplates of the dissolving and degradable MN arrays, and the entire hydrogel array, were removed, and the skin surface was cleaned once with a 70% isopropyl alcohol wipe. The same experimental setup and conditions (including Franz cell mounting and incubation at 32 °C) were employed prior to both the confocal Raman spectroscopy and SRS microscopy analyses. Control skin samples, which had not been treated with any of the MN arrays, were also examined in the same way.

### Confocal Raman spectroscopy

Confocal Raman spectroscopy was performed using a Renishaw RM1000 Raman microscope. A 1200-line/mm grating, which provided a spectral resolution of 1 cm^−1^, was used with a diode laser operating at 785 nm [14]. The Raman band (520 cm^−1^) of a silicon wafer was used as a calibration reference sample to correct potential Raman intensity changes or frequency shifts (daily calibration). The Live Track feature was used to maintain consistent imaging of uneven or curved surfaces for MN and skin sample characterisation. All Raman spectra were acquired in the fingerprint region from 800 to 1800 cm⁻^1^.

The reference spectra of all polymers (in solid form) were acquired to identify characteristic Raman bands that overlap, as minimally as possible, from those of the skin. Briefly, a few mg of each polymer were placed on aluminium foil and reference spectra were acquired using 4 accumulations (10 s each) with the laser operating at 100% power.

The distribution of each polymer within the MN array was also characterised. The array was positioned perpendicular to the direction of the objective lens, so that one needle could be targeted in the center of the white light image. Mapped measurements were performed with only 1 accumulation of 4- and 10-s exposure times, and a laser power of 100% (207 mW), for the dissolvable and degradable MNs, and for the hydrogel-forming MNs, respectively. In the case of dissolvable MNs, a 10 (x-axis) µm × 10 (y-axis) µm step size was used, scanning through a 30 (x-axis) µm × 190 (y-axis) µm square area (total 80 points). For degradable MNs, a 13 (x-axis) µm × 22 (y-axis) µm step size was used, scanning through a 39 (x-axis) µm × 484 (y-axis) µm square area (total 92 points). For hydrogel-forming MNs, a 12 (x-axis) µm × 27 (y-axis) µm step size was used, scanning through a 48 (x-axis) µm × 432 (y-axis) µm square area (total 85 points). Representative spectra from each MN base were acquired using 40 accumulations (10 s each) and 100% laser power. For the degradable MNs, representative spectra from the needle tip were also acquired.

MN-treated areas of skin were assessed to evaluate polymer distribution. The skin sample was placed in a custom-made sample holder and depth profiles were acquired ‘top-down’, every 50 µm, from the surface; 40 accumulations (20 s each) and a laser power of 100% were used with a long 50 × working distance objective lens [14]. The pores created by MN were visible under white light, and three were randomly selected for analysis. Untreated skin was examined as a control. The distribution of polymers within the skin was analysed by monitoring the intensities of PVP, PVA, Gantrez®, and PLGA at their characteristic wavenumbers: 752 cm⁻^1^ (C–C stretching) for PVP, 1728 cm⁻^1^ (C = O stretching) for PVA, 1716 cm⁻^1^ (C = O stretching) for Gantrez®, and 1768 cm⁻^1^ (C = O stretching) for PLGA. Amide I and phenylalanine signals from endogenous skin components were detected and recorded.

### Stimulated Raman scattering (SRS) microscopy

For top-down image acquisition, a double-sided adhesive tape spacer (Grace Bio-Labs, SecureSeal™, Sigma Aldrich, USA) was placed in the centre of a coverslip [14]. The treated skin sample was cut to smaller size and positioned within the circular opening of the spacer so that the *stratum corneum* was in direct contact with the coverslip. Another coverslip was then placed over the sample to minimise tissue dehydration during the experiment. The sample was secured on the stage with a clamp to prevent movement and to keep the sample from flexing. Throughout sample preparation and SRS imaging, the samples were maintained at room temperature. SRS and SHG microscopies were performed on a Leica SP8 laser scanning microscope (Leica Microsystems, Wetzlar, Germany) equipped with a PicoEmerald-S laser system (APE, Berlin, Germany). Images were acquired using a 25 ×/0.95 NA water immersion objective. SHG signals were epi-detected, while SRS signals were collected in transmission, using a 0.90 NA air condenser (Leica S1 50,515). The laser power used was 30% (36 mW and 11.7 mW at the sample for the Stokes and pump beams, respectively), which was selected to produce a satisfactory signal-to-noise ratio while minimising sample damage, including dehydration and shrinking. SRS image stacks were recorded from the skin/MN pore in triplicate from the tissue surface to a depth of 160 μm, at 20 µm intervals. The data acquisition parameters for top down included a line accumulation of 1, a scan speed of 200 Hz, and a line average of 1. The pump beam was tuned to achieve SRS contrast for CH_3_ stretching at 2945 cm^−1^. Off-resonance SRS signal contributions at 2600 cm^−1^ were also recorded to identify parasitic signals for subsequent subtraction (see data analysis section). SHG imaging was performed using the 797 nm excitation wavelength and detection at 398.5 nm using a Semrock FF01-414/46–25 BrightLine single-band bandpass filter.

Finally, as the sensitivity of SRS to detect polymer-specific signals as a function of depth is limited, cross-sectional SRS images of MN-treated skin samples using cryo-microtoming was developed. The MN-treated samples were immediately immersed in liquid nitrogen. This rapid cooling facilitated the easy removal of the baseplate. A Cryostat microtome (Leica CM1850, Wetzlar, Germany) was set to a sample temperature of −16 °C and blade temperature of −25 °C. The section thickness was set to 20 µm. Sections were thaw-mounted onto glass coverslips, lightly dried under argon gas, vacuum packed, and stored at −80 °C until analysis. These samples were analysed without a spacer and coverslip on top. The pump beam was tuned to achieve SRS contrast at 768 cm^−1^ for the C–C stretching vibration of PVP. Spurious signals were identified and removed by acquiring and subtracting the corresponding off-resonance frequencies at 2600 cm^−1^ and 702 cm^−1^ for the C-H stretching and fingerprint regions, respectively. Although the gain settings used in the fingerprint and C-H stretching frequencies were different, they were consistently applied during both the corresponding on- and off-resonance acquisitions. The workflow of this specific methodology is summarised in Supplementary Fig. S1.

### Data analysis

WiRE software (version 4.1) was used for confocal Raman spectral acquisition and pre-processing, including the removal of cosmic ray spikes (width parameter: 3, height parameter: 15) and baseline subtraction (polynomial order: 11). Maximum intensities of polymer and skin components were monitored via peak fitting (using the curve-fitting tool). The standard deviation of the noise was calculated by analysing the silent region in each spectrum: 1730–1800 cm⁻^1^, 1770–1800 cm⁻^1^ and 1740–1800 cm⁻^1^ were used for the dissolvable, degradable and hydrogel-forming MNs, respectively. The critical level of detection was determined as three times this standard deviation. The intensities of the MN samples, (i.e., from PVP, PLGA, and PVA) and of the skin components were measured in each spectrum, and the corresponding intensities from untreated skin were recorded for comparison. The direct classical least squares component analysis tool was used to assess the distribution of each polymer in the acquired MN maps. For each MN type, the reference spectrum of each polymer was used for comparison. Given the primarily qualitative objectives of this investigation, reference and map spectra were normalised with respect to the maximum intensity recorded (and were therefore scaled to values between 0 and 1). The distribution of each polymer in the map (correlation value of each reference spectrum to the MN spectrum) was then calculated. The latter indicates the degree of similarity (and is measured between values of 0 and 1) between a measured spectrum to a reference spectrum; the higher the value, the more the measured spectrum matches the reference. For the dissolving MNs, the reference spectrum of PVP K29/32 was used to acquire the distribution of both PVP grades present in the MN.

All SRS images were processed using ImageJ [19]. The “top-down” images were stacked as a function of depth. The grey scales of these z-stacks measured at the off-resonance control wavenumber were subtracted from the corresponding on-resonance values for all SRS signals acquired using the Image Calculator tool. It is noted that tissue movement (specifically in the z-direction) is expected as the sample shrinks from heat; however, image registration was not performed in the ‘top-down’ approach because the complexity of the skin structure results in non-uniform shrinkage and renders image alignment challenging. The processed image stacks were then colour-merged using the merge channel plugin for CH_3_ and SHG. A similar approach was followed to process the “cross-section” images. Large area mosaic-tile skin surface scanning, acquiring images of each tile at X1 zoom with an imaging speed of 400 Hz, was performed using LAS-X ‘Navigator’. The resulting image tiles were then stitched together using the ‘mosaic merge’ function in the Leica LAS-X software.

## Results

**Fig. 1:**
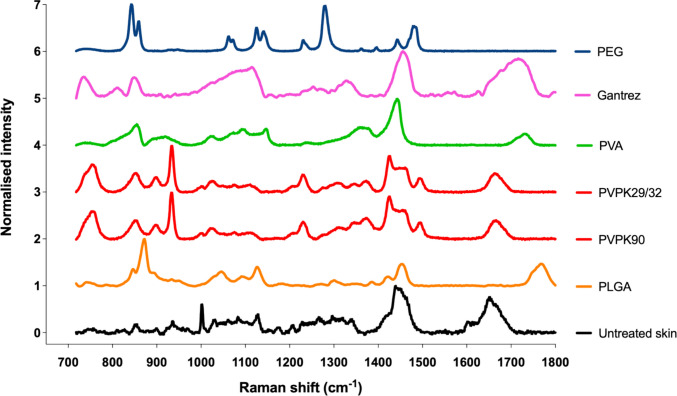
Reference Raman spectra of untreated skin, PLGA, PVPK29/32, PVPK90, PVA, Gantrez® and PEG. Spectra are normalised based on their corresponding maximum intensity

Raman spectra from the polymers used to fabricate the MN arrays were compared with one another and with that of untreated skin in an attempt to identify specific, interference-free peaks for each polymer (Fig. 1). However, the spectra of the two PVPs, PVPK90 and PVPK29/32, showed no distinguishable differences and the band at 752 cm^−1^ (corresponding to C–C stretching) was therefore used to track the disposition of both polymers. Carbonyl (C = O stretching) peaks at 1728, 1768 and 1716 cm⁻^1^ were assigned to PVA, PLGA and Gantrez®, respectively, but no peak for PEG could be found that did not overlap significantly from signals originating in the skin. As previous work shows, for Raman spectroscopy to successfully track the skin permeation or disposition of a chemical (drug and excipients), the latter must have a molecular vibrational signal that lacks interference from those emanating from endogenous skin species [14]. It was also noted that, for the other polymers, a degree of overlap with both the endogenous Amide I signal (1666 cm^−1^) from the skin (primarily from keratin), and with a sharp phenyl ring breathing signal (often assigned to phenylalanine at 1003 cm^−1^), was evident.

### MN mapping using confocal Raman microscopy

**Fig. 2:**
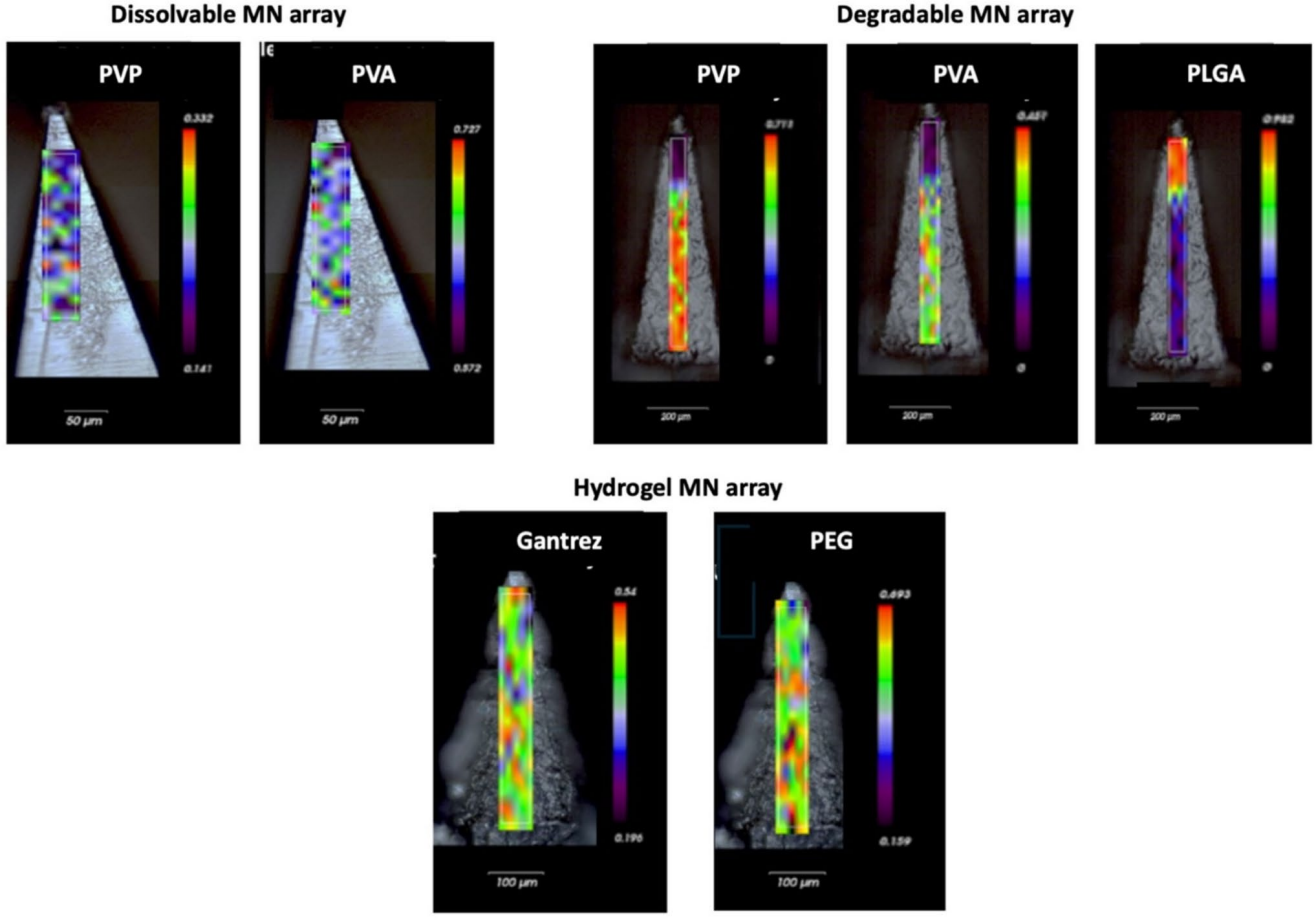
Raman mapping component analysis to show the distribution of polymers in the different MN arrays. The panels illustrate the coloured polymer heat maps (i.e., the measured signals normalised by the reference spectrum of the corresponding polymer) superimposed on the white light images of the MNs (in grey). Colours correspond to the correlation value of each reference spectrum to the MN one. Both PVPK90 and PVPK29/32 signals contribute to the component analysis for the dissolvable MNs, whereas only that from PVPK29/32 contributes to that of the degradable MNs. A single needle from each MN array was used for the polymer heat map for each of the component polymers of the respective arrays

The confocal Raman maps characterising the distribution of each polymer in the different MN arrays are shown in Fig. 2. The MN spectra represent a complex combination of those of the individual polymers present in each array. Component analysis was able to separate the MN components and demonstrate that their distribution in the dissolvable and hydrogel-forming arrays was relatively homogeneous. In contrast, the polymers in the degradable MN arrays were spatially differentiated with PLGA located primarily in the tips, and PVP and PVA in the central/lower parts of the MNs.

### Polymer distribution in skin after MN insertion

#### Dissolvable MNs

**Fig. 3:**
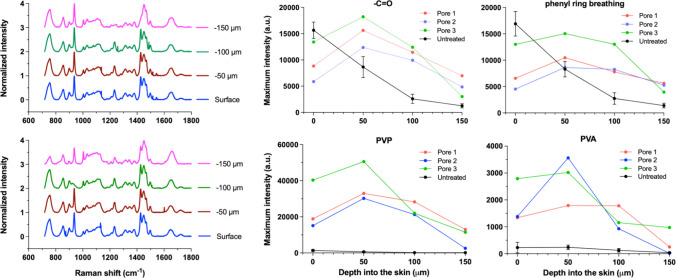
Left panels: Raman spectra after insertion of dissolvable MN arrays of the skin surface and at depths of 50 μm, 100 μm and 150 μm in two pores. Upper graphs: -C = O and phenyl ring breathing signals (mean ± standard deviation) in three dissolvable MN-created pores at the skin surface (n = 3 measurements) and at the three skin depths considered. Lower graphs: The corresponding signals from the PVP (both PVPK90 and PVPK29/32 combined) and PVA polymers from which the dissolvable MN arrays were made

Fig. 3 (left panels) shows two illustrative series of Raman spectra as a function of depth into the skin following insertion of the dissolvable MN arrays. These spectra were recorded at clearly identifiable pores in the skin post-insertion of the microneedles at nominal depths of 50 µm, 100 µm and 150 µm; for comparison, corresponding spectra were also acquired from untreated, control skin.

Signal intensities from three MN-created pores in the skin (and again compared to the control, untreated tissue) at the skin surface and at the three aforementioned depths are shown in the remaining four panels of Fig. 3. The upper two graphs show the -C = O and phenyl ring breathing signals that are always present in the skin’s Raman spectrum. In untreated skin, these signals are attenuated with increasing depth due to scattering and absorption of the radiation by the tissue. In MN-porated skin, however, this attenuation is noticeably altered and, in the pores studied, did not fully return to ‘normal’ even at 150 µm. At the same time, Raman signals from PVP and PVA (the lower two graphs in Fig. 3) are clearly identifiable in the pores, only falling away at the deepest skin depth considered; in contrast, there is as expected no evidence of the polymers in untreated skin.

**Fig. 4:**
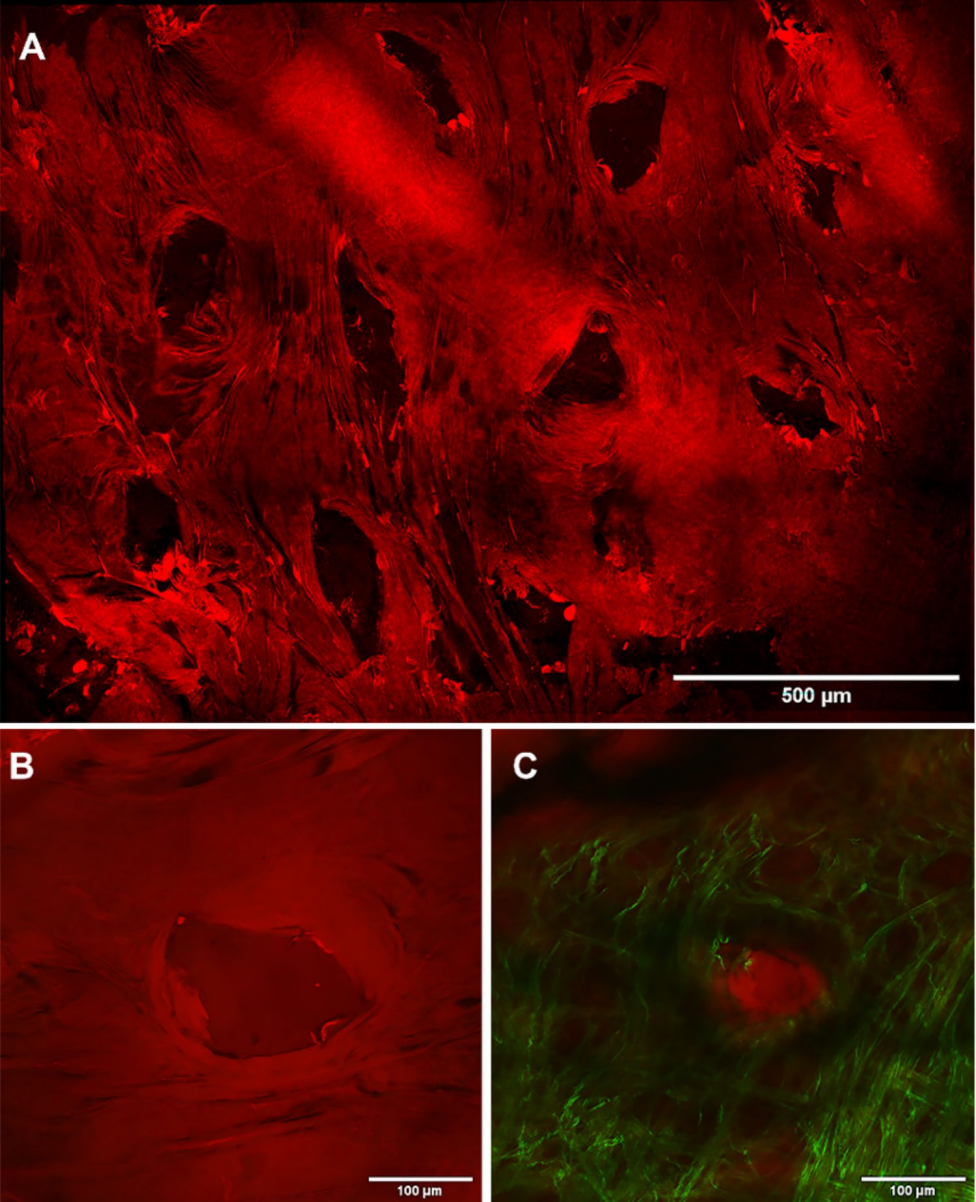
SRS images of the skin surface post-treatment with a dissolvable MN array. **A** Large-area mosaic image showing the regular pattern of pores created. **B** Close-up of one such MN-created pore. **C** SRS image of the same pore recorded at 50 µm beneath the skin surface. The SRS signal from -CH_3_ stretching at 2945 cm^−1^ is shown in red; SHG, which is typically associated with collagen, is highlighted in green

Figure 4A shows a large area, mosaic SRS image at 2945 cm^−1^ (-CH_3_ stretching vibration (red) predominantly from proteins) of the skin surface post-insertion of the MN array – the dark, approximately circular features of the MN-created pores are clearly visible; Fig. 4B shows a close-up of one such pore. Images taken progressively deeper into the skin (Supplementary Information Fig. S2 and Movie S1) demonstrate the extent to which the MNs penetrate the tissue and allow sub-surface structural changes to be identified from the SHG signals recorded (Fig. 4C). It is noted that these signals appear at shallower depths than anticipated because the MN application process compresses the skin and, due to the unavoidable skin dehydration during SRS measurements, epidermal contraction occurs.

#### Degradable MNs

**Fig. 5:**
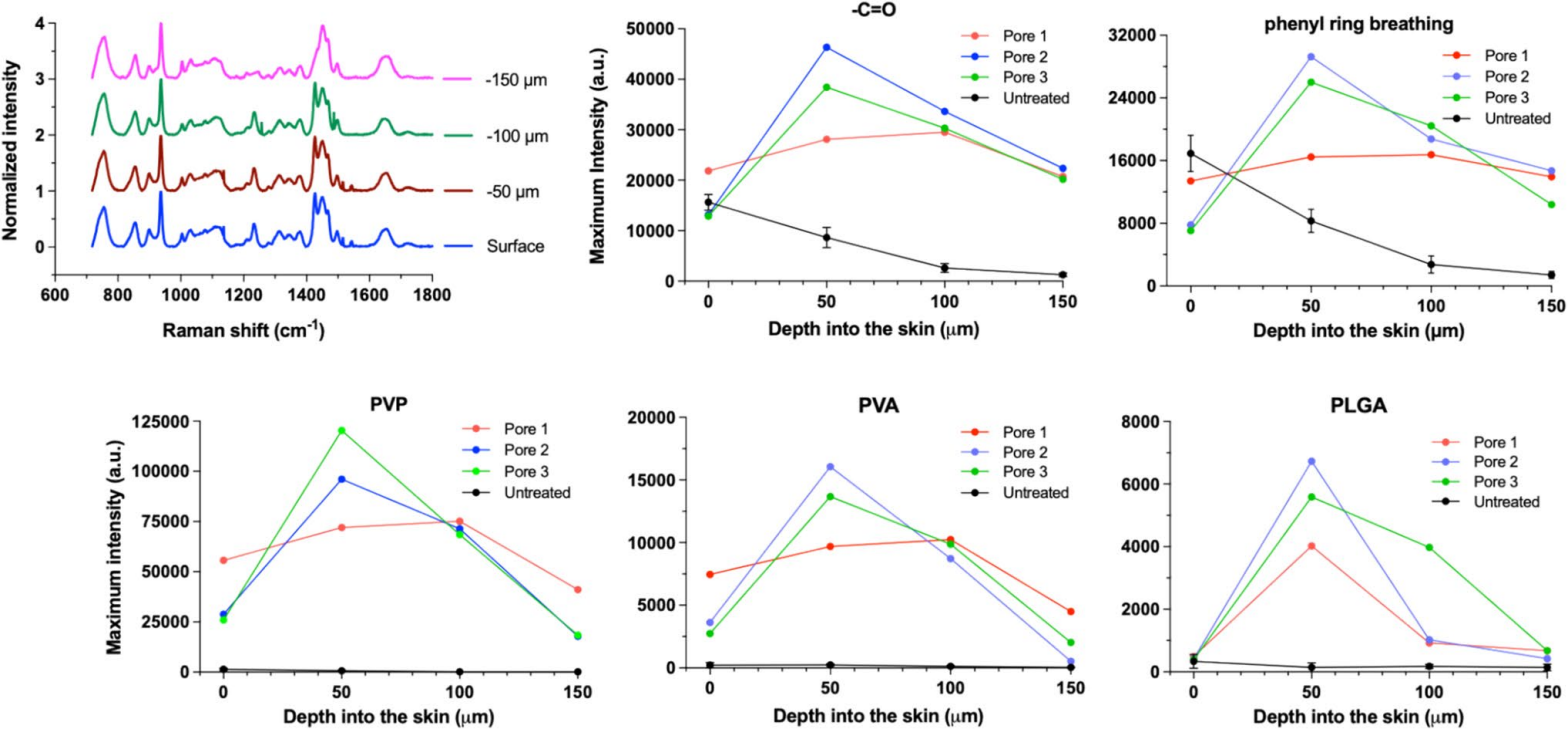
Upper left panel: Raman spectra after insertion of degradable MN arrays of the skin surface and at depths of 50, 100 and 150 μm in two pores and in untreated (non-porated) tissue. Upper graphs: -C = O and phenyl ring breathing signals (mean ± standard deviation) in three degradable MN-created pores at the skin surface (n = 3 measurements) and at the three skin depths considered. Lower graphs: The corresponding signals from the PVP (both PVPK90 and PVPK29/32 combined), PVA and PLGA polymers from which the degradable MN arrays were made

Figure 5 (top left panel) shows representative Raman spectra as a function of skin depth after insertion of the degradable MN array. As before, the spectra were acquired at obviously discernible pores in the skin after microneedle treatment at nominal depths of 50 µm, 100 µm and 150 µm; again, comparative spectra were recorded from untreated skin

Raman signals from three MN-created pores (plus untreated controls) at the skin surface and at 50, 100 and 150 µm are shown in the other five panels of Fig. 5. The upper graphs present the endogenous -C = O and phenyl ring breathing signals. The normal attenuation of these signals in untreated skin is again clearly different in MN-porated skin, in particular at 50 and 100 µm. In parallel, Raman signals from the polymers comprising the degradable MN arrays (the lower three graphs in Fig. 5) are visible in the pores, and well above the critical level of detection to a depth of 100 µm; once more, there were no significant signals detected at the polymer wavenumbers in the untreated skin.

**Fig. 6:**
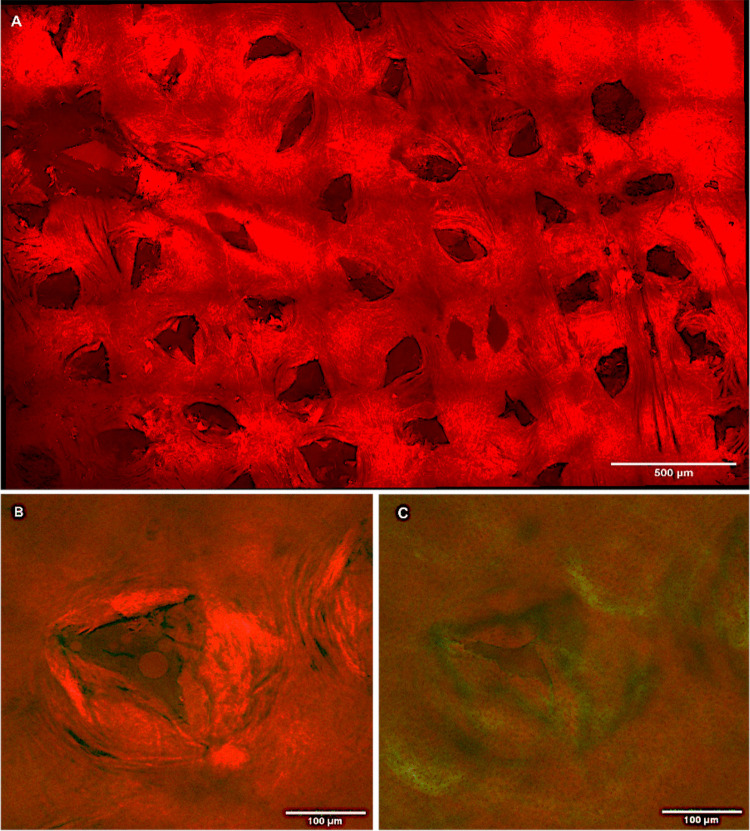
SRS images of the skin surface post-treatment with a degradable MN array. **A** Large-area mosaic image showing the regular pattern of pores created. **B** Close-up of one such MN-created pore. **C** SRS image of the same pore recorded at 50 µm beneath the skin surface. The SRS signal from -CH_3_ stretching at 2945 cm^−1^ is shown in red; SHG, which is typically associated with collagen, is highlighted in green

Figure 6A is a large, mosaic SRS image at 2945 cm^−1^ (-CH_3_ stretching vibration (red) principally from lipids/proteins) of the skin surface after MN application – the microneedle-created pores are easily recognisable as the regularly spaced dark features; a zoomed-in image of a single pore is shown in Fig. 6B. Supplementary Information Fig. S3 and Movie S2 presents images taken progressively deeper into the skin showing the degree of penetration of the MNs into the tissue and permitting structural alterations to be deduced from the SHG signals as before (Fig. 6C). Again, as for the dissolvable MN arrays, the specific depths at which these signals are seen may be under-estimated.

#### Hydrogel-forming MNs

**Fig. 7:**
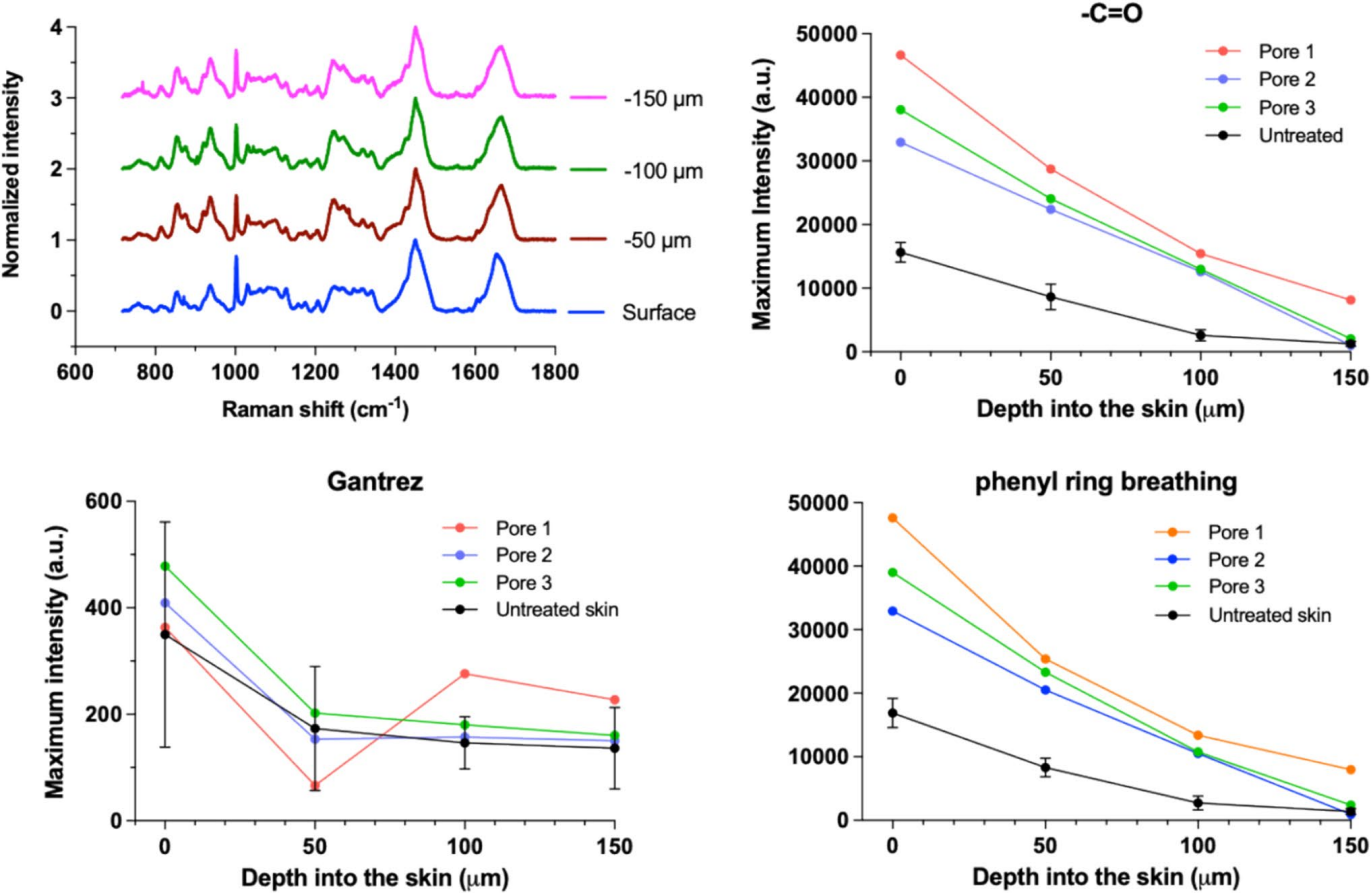
Top left panel: Raman spectra after insertion of hydrogel-forming MN arrays of the skin surface and at depths of 50 µm, 100 µm and 150 μm in a single pore. Right graphs: -C = O and phenyl ring breathing signals (mean ± standard deviation) in three hydrogel-forming MN-created pores at the skin surface (n = 3 measurements) and at the three skin depths considered; the corresponding results from untreated skin are shown for comparison. Lower left graph: The corresponding signals from the Gantrez® polymer from which the hydrogel-forming MN arrays were made

Figure 7 summarises the confocal Raman results after insertion of the hydrogel-forming MN array; the upper left panel shows Raman spectra collected from a single pore at the surface, and then at depths of 50 µm, 100 µm and 150 µm, after removal of the microneedles. Under the experimental conditions used, and as expected, Gantrez® was not detected given that these hydrogel-forming MN arrays post-removal are designed to leave no residual polymer in skin.

**Fig. 8:**
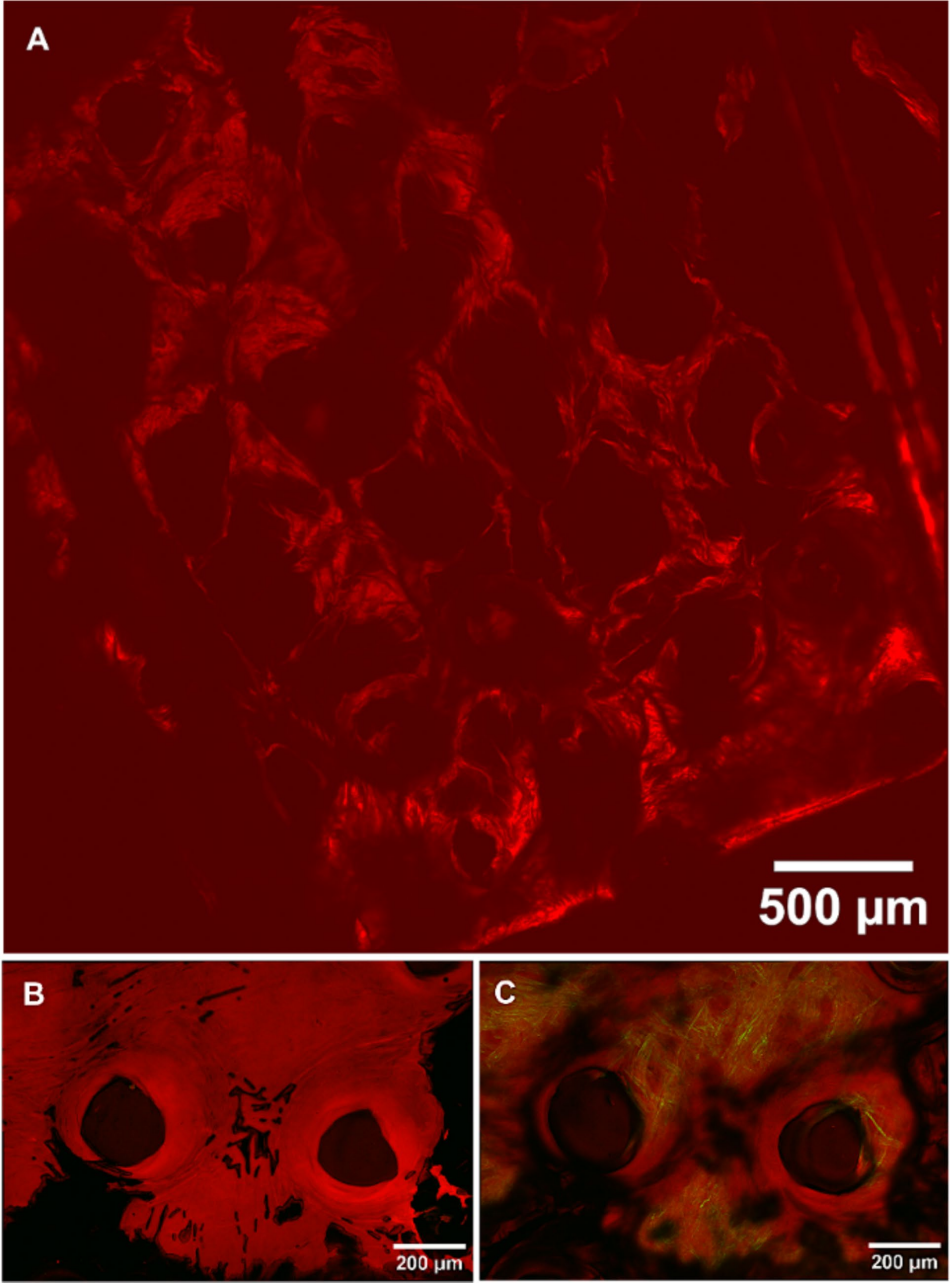
SRS images of the skin surface post-treatment with a hydrogel-forming MN array. **A** Mosaic image showing creation of a regular pattern of pores. **B** Close-up of two pores acquired at the skin surface. **C** SRS image of the same pores at 50 µm beneath the skin surface. The SRS signal from -CH_3_ stretching at 2945 cm^−1^ is in red; SHG, which is typically associated with collagen, is in green

Figure 8A is a large-scale, mosaic SRS image at 2945 cm^−1^ of the skin surface after hydrogel-forming MN array insertion and removal; the regular pattern of the pores created by the microneedles is evident. Figure 8B and C are respectively magnified images of two individual pores acquired at the skin surface and at a depth of approximately 50 µm below. Supplementary Information Fig. S4 and Movie S3 presents a series of images taken as a function of depth into the skin and illustrates the eventual appearance of SHG signals (most likely from collagen) indicating that the MN array had porated the skin into the dermis.

### SRS imaging of skin cross-sections post-MN insertion

Snap-freezing of the skin and cross-sectioning was performed immediately after application of MN to preserve sample structure. The focus was to detect PVP, the polymer with the highest signal intensities measured with confocal Raman.

**Fig. 9:**
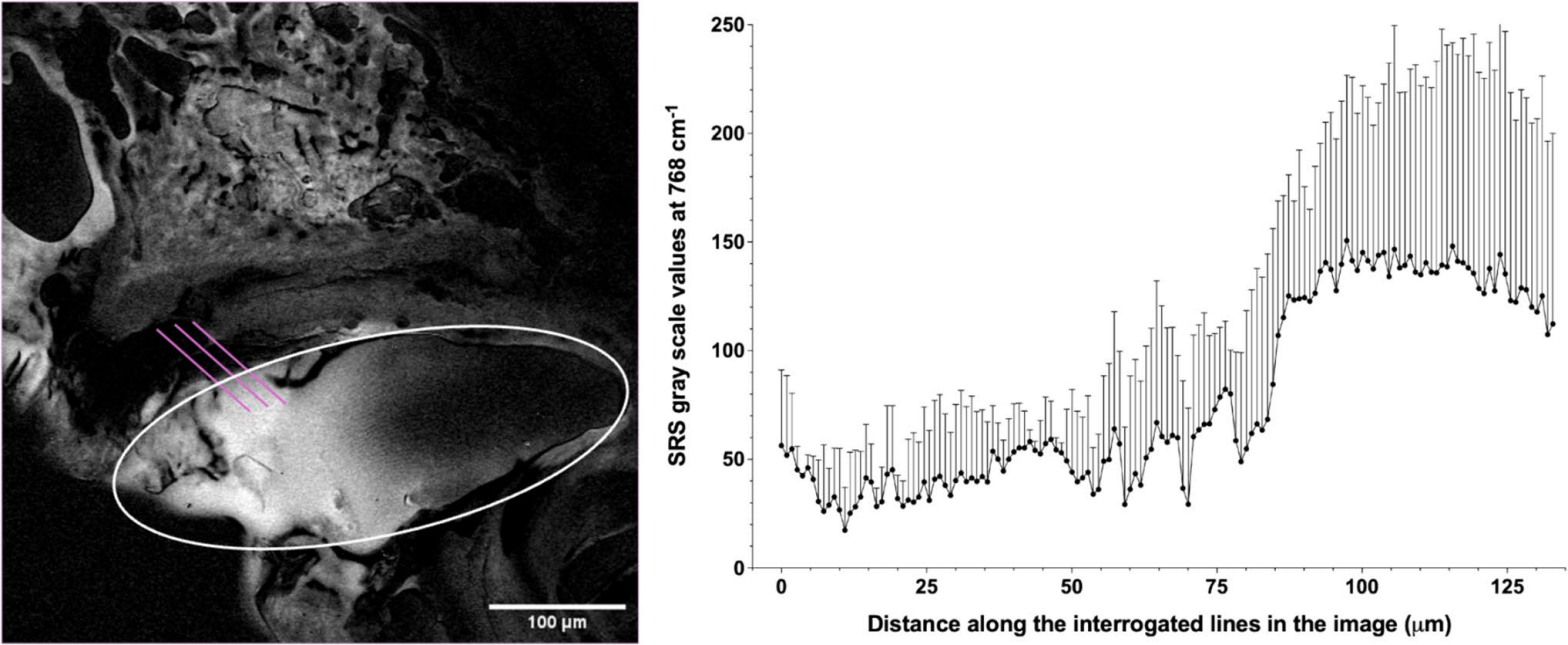
Left panel: SRS image of a MN-created pore (circled) in a cross-sectioned skin slice of 20 µm thickness; the imaged band is 768 cm^−1^. Right panel: The SRS grayscale intensity values at 768 cm^−1^ recorded as a function of distance along the three magenta lines in the image at left transitioning from unperturbed skin into the MN-created pore. Data are mean + SD, *n* = 3

A representative cross-sectional image of a dissolvable MN-treated skin sample shows clearly the insertion of the array into the barrier (Fig. 9**, left panel**). The imaged Raman band at 768 cm⁻^1^ captures signals originating from both endogenous skin species and from the PVP present in the array. This is illustrated in the SRS grayscale values collected at this frequency (Fig. 9**, right panel)** as data are acquired along the three magenta lines in the image to the left. The upper approximately 2/3 of the lines records the 768 cm⁻^1^ signals from the skin but these then increase appreciably as the lines descend into the MN-occupied pore, reflecting a new and important contribution from the polymer.

## Discussion

The characterisation of MN arrays has evolved over the past 25 years. One critical aspect, of course, is the consistency of needle insertion into the skin, the real-time monitoring of which has proved to be a challenging task. The techniques considered to-date include optical coherence tomography (OCT), X-ray tomography, and confocal microscopy [8, 9, 20]. The latter requires the use of fluorescent markers or tags and remains limited with respect to depth resolution, which has typically been restricted to the outermost skin layers, and application of X-ray tomography has been used insufficiently to properly judge its potential. OCT, on the other hand, has proven to be a valuable tool, providing real-time imaging *ex vivo* and *in vivo*. Here, confocal Raman spectroscopy and SRS microscopy have been applied to the assessment of MN array insertion into the skin, and the skin disposition of the polymeric constituents of dissolving, degradable, and hydrogel MN arrays.

With the exception of PEG, it was possible to identify distinct, and mostly interference-free, Raman signals for each of the polymers used to create the different types of MN arrays studied in this research (Fig. 1). This is clear from the first set of experiments that characterised the polymers’ distribution in the microneedles post-fabrication. For the dissolvable and hydrogel-forming arrays, the constituent polymers were relatively evenly distributed within the MNs (Fig. 2). In contrast, for the degradable MNs, PLGA was found only in the needle tips, while PVP and PVA were co-located in the rest of the array; this is the desired distribution with the intention that the MN tips remain in the skin post-removal of the array slowly degrading to controllably release the drug therein [17].

A series of confocal Raman experiments were then performed to determine whether the MN-associated polymers’ distribution within the skin post-application *ex vivo* could be characterised. To maximise the consistency of MN insertion, the skin was carefully stretched and secured with thumb tacks to mitigate the impact of the tissue’s inherent elasticity and to minimise its movement during the procedure. While the approach may not precisely replicate application of a MN array *in vivo*, the goal was to optimise insertion repeatability and depth as far as possible and to ensure and acquire consistent polymer detection and analysis across the skin samples examined.

Untreated skin shows endogenous Raman signals at 1666 and 1003 cm^−1^ originating form endogenous Amide I (primarily keratin) -C = O vibration and phenyl ring breathing (often attributed to phenylalanine [14, 21], respectively. Both of these signals decayed as a function of depth into the skin due to absorption and scattering as previously reported [21]. Post-treatment with both the dissolvable and degradable MN arrays, the Raman signals at 1666 cm^−1^ and 1003 cm^−1^ were clearly enhanced at nominal depths of 50 µm and 100 µm due to significant overlap from those of PVP and PVA (Figs. 3 and 5) – and, in the case of the degradable MNs, PLGA as well. A plausible hypothesis to explain these observations is that, post-MN insertion into the skin, residual polymers in the pores are responsible for the increased signals recorded at least until a depth of 150 µm where the intensities return to those of the untreated control values, reflecting the point at which any meaningful contribution – over and above that from the endogenous skin components – is reached. Further support for this interpretation of the data is that post-MN insertion, the peak of the “Amide I” signal was located, especially up to 100 µm into the skin, at 1657 cm^−1^ (as opposed to 1666 cm^−1^) suggesting contributions from PVP/PVA to the detected -C = O vibration.

For the hydrogel-forming MNs, no residual polymer was expected post-removal of the arrays and, indeed, the signals at 1666 cm^−1^ and 1003 cm^−1^ attenuated progressively as a function of depth like the untreated control. However, the signal intensities observed were noticeably higher and this is possibly due to the effect of compression of the skin during MN insertion effectively causing an apparent increase in the ‘concentration’ of Amide I/phenylalanine within the focal volume of the instrument’s laser. It is also conceivable that – while the intention was to focus the laser on the centre of the pore – that contributions to the signals detected were additionally acquired from adjacent areas of the (compressed) skin.

While the relatively slow acquisition time of the confocal Raman means that only a limited number of the MN-created pores can be examined due to dehydration of the skin and the consequent deterioration in data quality, SRS microscopy offers rapid and high-resolution depth imaging of relatively large areas of the tissue. The multi-tile, mosaic scans possible with SRS (Figs. 4, 6 and 8) enable visualisation of the microneedle-created pores in the skin and reveal the complex topology of the evidently non-flat nature of its surface; indeed, the extent of this inhomogeneity is readily apparent in the lower right-hand corners of Figs. 4A and 8A (below the scale bars) which show that the skin surface in these areas was not in contact with the bottom coverslip used.

However, the ability to scan quickly over larger areas comes with the ‘price’ of reduced sensitivity to detect polymer-specific signals ‘top-down’ with the SRS as a function of depth. To address this limitation, an approach was developed to acquire cross-sectional SRS images of MN-treated skin samples using cryo-microtoming to preserve tissue structure and to demonstrate detection of PVP from the microneedles (Fig. 9). By scanning along lines originating outside an MN-created pore, then across the ‘rim’ of the pore, and finally into the pore itself (Fig. 9, left panel), there is a clear step-change in the signal at 768 cm^−1^ (Fig. 9, right panel) demonstrating – within the pore itself – an important contribution from residual polymer over and above the background from endogenous skin species.

## Conclusion

This research successfully addressed the two main objectives of the project: (i) to assess polymer presence and distribution in the skin after microneedle (MN) application using confocal Raman spectroscopy, and (ii) to evaluate MN insertion depth and uniformity with SRS microscopy. Confocal Raman spectroscopy enabled polymer detection without tissue sectioning or staining and did not visibly damage the skin. SRS imaging revealed consistent penetration patterns, although insertion depth inevitably varied due to skin surface inhomogeneity and roughness. Together, these vibrational spectroscopic techniques confirmed that MNs create well-defined microchannels through the *stratum corneum* and epidermis into the dermis. The complementary strengths of confocal Raman (chemical characterisation) and SRS microscopy (spatial resolution with high speed) offer robust and non-invasive tools with which to study MN penetration dynamics. Future work will focus on the fate of MNs post-insertion and the assessment of the safety of repeated MN array application, which are key questions that must be answered to advance the development and regulatory acceptance of this drug delivery technology.

## Supplementary Information

Below is the link to the electronic supplementary material.

Supplementary file1 (DOCX 2938 KB)

Supplementary file2 (AVI 345 KB)

Supplementary file3 (AVI 523 KB)

Supplementary file4 (AVI 2065 KB)

## Data Availability

Materials used are either directly available from the providers or their formulation described in methods. The datasets generated during and/or analysed during the current study are available from the corresponding author on reasonable request.
