# Supplementary material for: Assessment of microneedle array insertion into skin using Raman spectroscopic techniques
Source: Drug Deliv Transl Res. 2026 Jan 13;16(8):2976–89. doi: 10.1007/s13346-025-02041-1 (PMC13346314; doi:10.1007/s13346-025-02041-1)
Supplement: Supplementary file 1 — Supplementary file1 (DOCX 2938 KB) [file 13346_2025_2041_MOESM1_ESM.docx]

**Supplementary Information**

**The Assessment of Microneedle Array Insertion into Skin using Raman Spectroscopic Techniques**

Rezvan Jamaledin^1^, Panagiota Zarmpi^1,2^, Adrián M. Alambiaga-Caravaca^1^, Vasundhara Tyagi^3^, Qonita Kurnia Anjani^4^, Eneko Larrañeta^4^, Ryan F. Donnelly^4^, Natalie A. Belsey^2,3^, Richard H. Guy^1^ & M. Begoña Delgado-Charro^1,*^

^1^ University of Bath, Department of Life Sciences, Bath BA2 7AY, UK.

^2^ University of Surrey, School of Chemistry & Chemical Engineering, Guildford, GU2 7XH, UK

^3^ National Physical Laboratory, Teddington TW11 0LW, UK.

^4^ School of Pharmacy, Queen’s University Belfast, Medical Biology Centre, Belfast BT9 7BL, UK.

* Correspondence: [prsbd@bath.ac.uk](mailto:prsbd@bath.ac.uk)


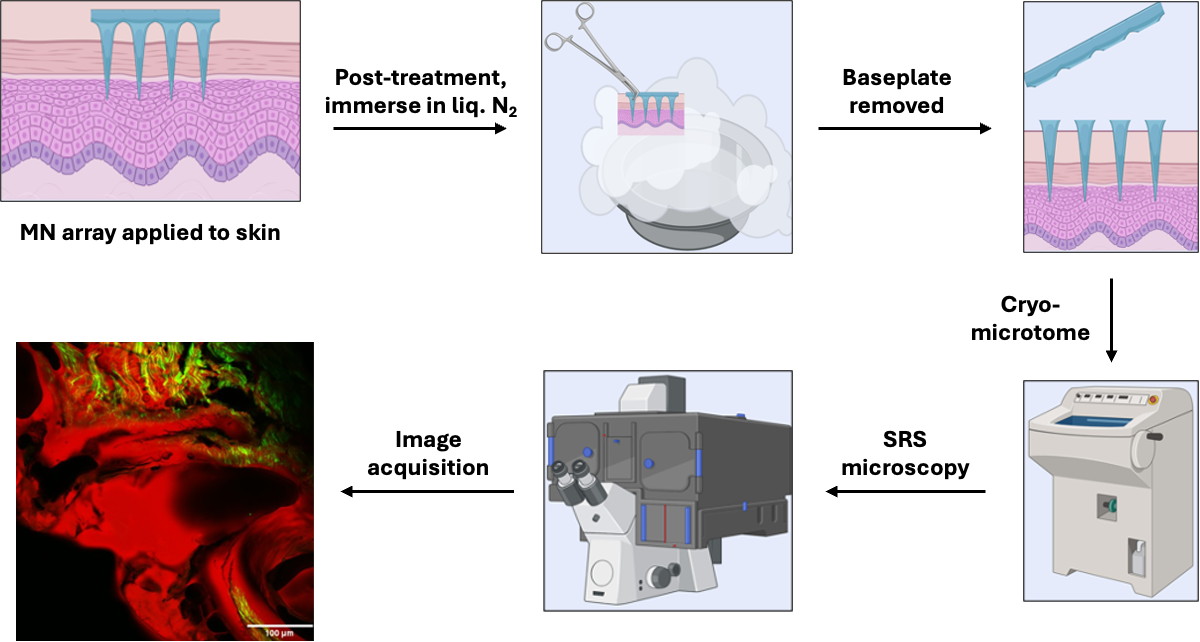


****Fig.S.1**. Workflow employed to acquire SRS imaging of cryo-microtomed cross sections of dissolvable MN-treated skin.**


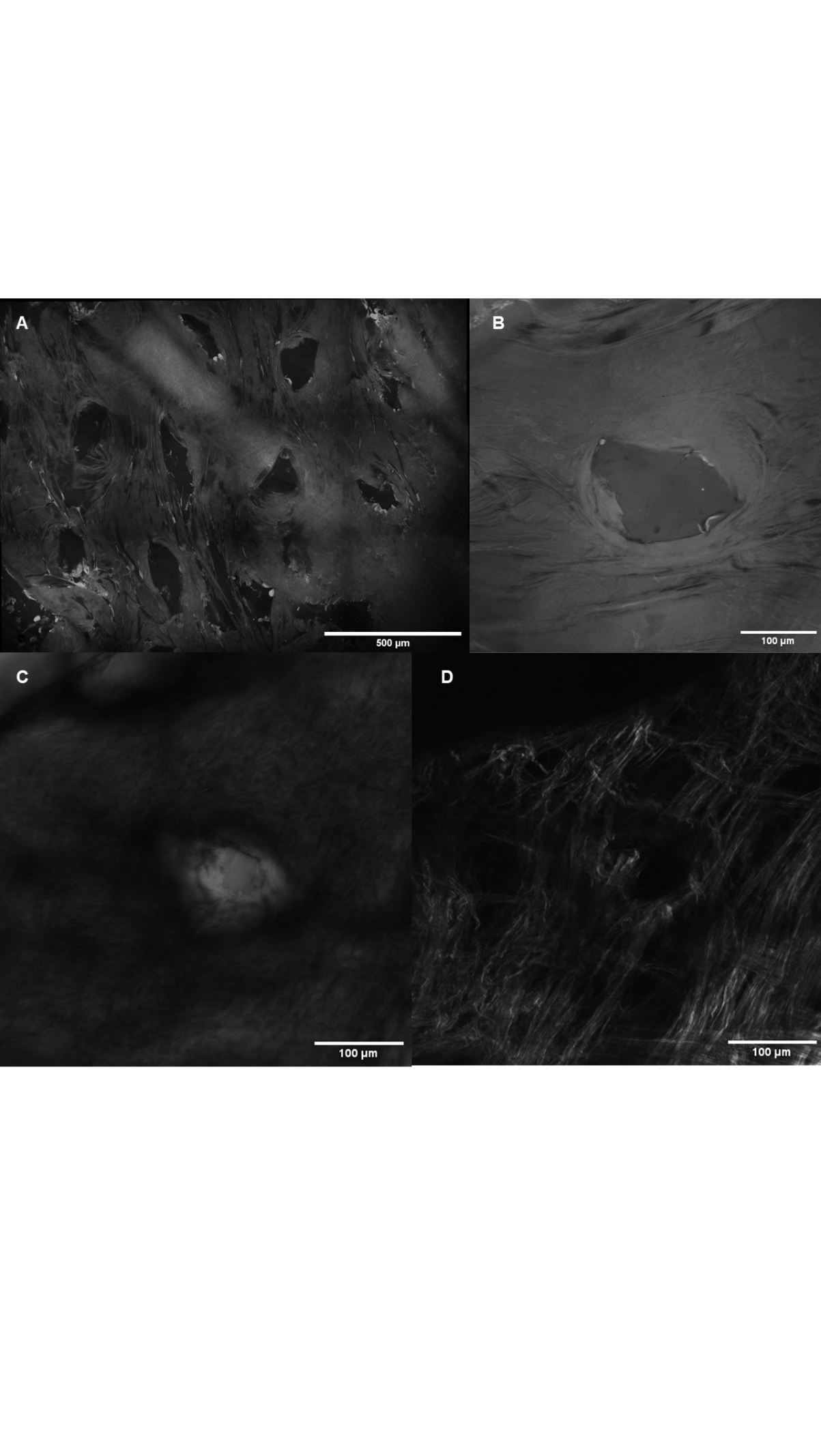


**Fig.S.2** SRS imaging of skin treated with the dissolvable MN array. A) Large area SRS mosaic image showing the regular pattern of pores created by a MN array. The resulting image tiles corresponded to areas of 465 μm × 465 μm. B) Close-up of one MN pore C) -60µm beneath it. D) Collagen signal at depth of -60 µm.


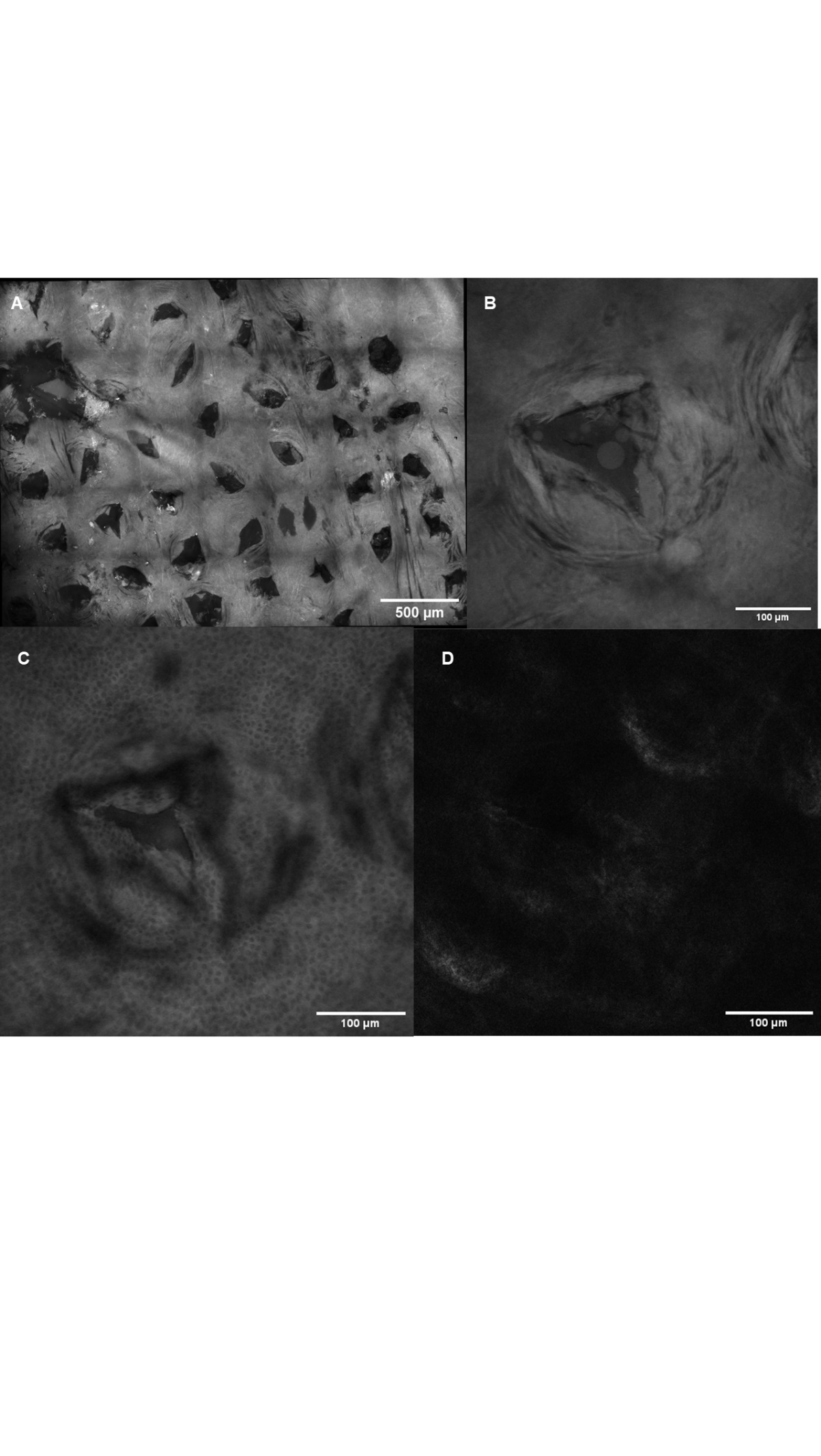


**Fig.S.3** SRS imaging of skin treated with the degradable MN array. A). Large area mosaic SRS image showing the regular pattern of pores created by a MN array. The resulting image tiles corresponded to areas of 465 μm × 465 μm. Close-up of one MN pore at B at 2945 cm^-1^ and C) -40 µm. D) Collagen signal at depth of -40 µm.


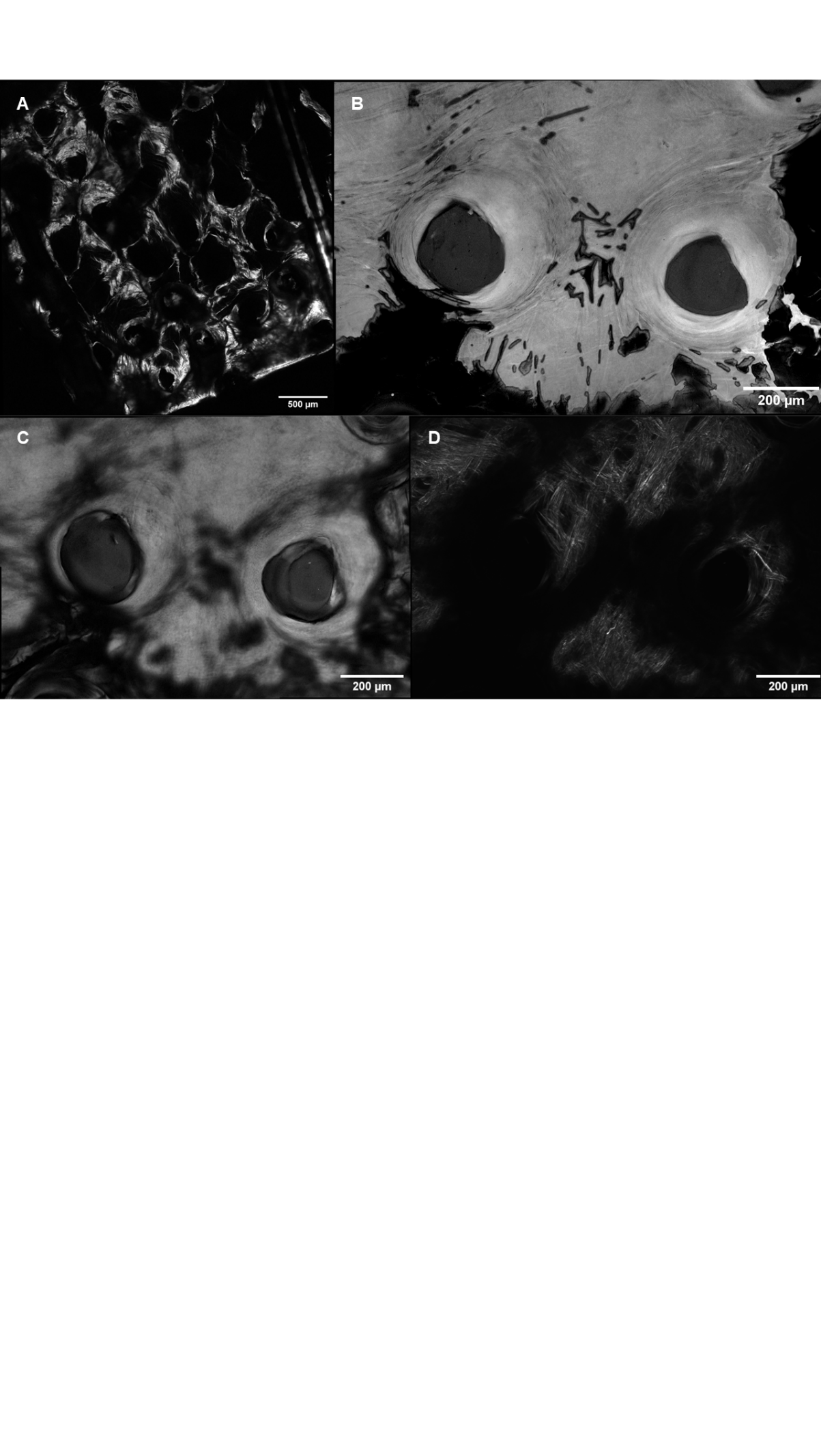


**Fig.S.4** SRS imaging of skin treated with the hydrogel MN array. A) Large area mosaic SRS image showing the regular pattern of pores created by a MN array. B) Close-up of two pores C) -60µm beneath it. D) Collagen signal at depth of -60 µm. Images acquired at 2945 cm^-1^.

**Movie S.1:** “Top-down” SRS image stacks from treated skin by dissolvable MNs showing the distribution of CH3 in red and the SHG in green.

[dissolvable.avi](file:///D:\University%20of%20Bath\Skin%20group\MN%20project\Paper%201\dissolvable.avi)

**Movie S.2:** “Top-down” SRS image stacks from untreated skin by degradable MNs showing the distribution of CH3 in red and the SHG in green.

[degradable.avi](file:///D:\University%20of%20Bath\Skin%20group\MN%20project\Paper%201\degradable.avi)

**Movie S.3:** “Top-down” SRS image stacks from treated skin by hydrogel MNs showing the distribution of CH3 in red and the SHG in green.

[hydrogel.avi](file:///C:\Users\rj832\Dropbox\Bath%20projects\Paper%201%20CR%20SRS\hydrogel.avi)
